# Adaptation among aged care and disability service providers in response to the COVID-19 pandemic: Lessons for the future

**DOI:** 10.3389/frhs.2022.1037256

**Published:** 2022-11-04

**Authors:** Ami Seivwright, Lisette Kaleveld, Ariella Meltzer, Mariana Atkins, Meera Varadharajan, Perri Campbell, Zoe Callis, Erin Wilson

**Affiliations:** ^1^Institute for Social Change, University of Tasmania, Hobart, TAS, Australia; ^2^Centre for Social Impact, The University of Western Australia, Perth, WA, Australia; ^3^Centre for Social Impact, University of New South Wales, Sydney, NSW, Australia; ^4^Centre for Social Impact, Swinburne University of Technology, Hawthorn, VIC, Australia

**Keywords:** aged care, disability service, COVID-19, organizational adaptation, client-centered care

## Abstract

Aged care and disability service organizations are critical infrastructure. However, in 2020, restrictions were introduced to reduce the infection risk of the coronavirus disease 2019 (COVID-19), and these organizations needed to quickly devise COVID-safe ways of working to continue to meet the needs of their clients. To investigate how these organizations adapted their service delivery and which innovations they felt were worthwhile for sustaining beyond the COVID-19 pandemic, interviews were undertaken with representatives from 26 aged care and disability service organizations across three states in Australia (Western Australia, New South Wales, and Victoria). Findings revealed that organizations adapted their practices across three key innovation areas: (1) developing new approaches or expanding existing services, particularly around food provision, social connection, information dissemination, and technology support; (2) modifying the mode of service delivery, through safe in-person contact or offering alternative online services; and (3) reducing bureaucracy and introducing remote working. A common theme across all service innovations was the strong focus on providing clients and staff with choice and control. Moving forward, many organizations wanted to integrate and maintain these innovations, as they were associated with additional benefits such as increased client health and safety, service flexibility, and sufficient human resources to serve clients. However, continued maintenance of some initiatives require additional resourcing. The continuation of COVID-19 pandemic adaptations and, indeed, ongoing innovation, would therefore be facilitated by greater flexibility of funding to allow organizations and their clients to determine the service types and modes that best meet their needs. Further, these innovations have implications for sector-wide best practice.

## Introduction

In Australia, as elsewhere, the coronavirus disease 2019 (COVID-19) pandemic significantly impacted the capacity for aged care and disability service organizations to continue to support the health and well-being of those relying on their services. Given the disproportionate risk and impacts of the pandemic on older people and those with disability, the impetus and stakes for innovation were high for organizations that seek to meet the physical health and other needs of these groups. In this qualitative study we present an overview of the service adaptations and innovations implemented by aged care and disability service organizations across three Australian states: Western Australia (WA), New South Wales (NSW), and Victoria (VIC). Further, we investigate which innovations organizations intend to retain beyond the COVID-19 pandemic and what they require for this continuation. For the purposes of the current study, when we refer to the COVID-19 pandemic, we are referring to both the health implications of COVID-19 itself, and the associated government-imposed protective measures that were put in place during 2020.

From the outset of the pandemic, there were concerns that social inequities would deepen. It was predicted that people with limited economic resources would be poorly placed to avoid infection and the resulting health consequences (e.g., by being less likely to have jobs that can be worked from home, less likely to have access to housing where they can safely isolate, and less able to “lock down” without suffering economic detriment), and consequently poorly positioned to weather the subsequent social and economic storm (1, 2). Additionally, not all pandemic responses were uniformly available to all. For example, with the expanded use of digital technologies, such as telehealth, came questions about its appropriateness and inclusivity (3). The lack of digital infrastructure and accessible information, limited digital literacy, lack of private spaces (e.g., in congregate residential settings), and poor accessibility of the technology used (e.g., for people with visual or cognitive impairment) acted as instrumental barriers to the receipt of technologically-mediated health services for some groups such as people with disability and those living in aged care (4).

In the context of people living with disabilities, in addition to concerns about the inclusivity and accessibility of digital health, there have been broader concerns that the COVID-19 pandemic could lead to regression from the social model of care that prioritizes individual rights and preferences, back to an institutionalized, medical model of care (5). In disability service organizations, exacerbating the risk of moving away from individual rights is what Doyle and O'Brien (6) termed the “cacophony of protocol” that has escalated during the pandemic. This comprises increased administrative and regulatory burdens, everchanging and often conflicting guidelines, and significant organizational risk if interpretation and implementation of guidelines are deemed “incorrect” after the fact. Accordingly, the increase in health needs and the “top down” management of disability services (i.e., by government) due to the public health responsibilities brought about by the pandemic introduced additional challenges for organizations in attempting to balance physical health and social needs of service users.

Aged care has also faced significant scrutiny throughout the pandemic. A substantial proportion of COVID-19 deaths have been people living in residential aged care facilities, attributed to a combination of individual factors, such as the tendency for older people to be more vulnerable to severe disease, people having comorbidities and complex care needs, and external factors, such as the design of facilities (7). “Lockdowns” of residential aged care facilities and restrictions to community care provision (e.g., reduced length of visits, fewer services offered) were implemented to reduce the risk of fatalities, however these actions also raised significant concerns about loneliness and social isolation, and their detrimental health effects, among older people (8). Much like disability service organizations, aged care organizations have had to balance the physical health and social needs of the people they serve. In Australia, the Royal Commission into Aged Care Quality and Safety, which had begun prior to the pandemic and continued into early 2021, revealed serious concerns about the quality of both health and social care received in Australia's aged care facilities. Alongside the need for stronger governance and increased funding of aged care services, key findings pointed to the need for improved workforce conditions and capability (9).

Workforce challenges during the pandemic have had a significant impact on the aged care and disability service sectors. The structure of the workforce, characterized by the high prevalence of casualisation, fee-for-service funding and the resultant “gig economy,” has led to concerns about a “collision course” in which workers transmit COVID-19 from client to client, even across separate services and settings. Risk mitigation (e.g., through increased infection control training) has been hampered by the decentralized nature of the workforce (8, 10). Indeed, there is evidence that some of these concerns were playing out early in the pandemic, with a report on disability support workers finding that workers were providing their own personal protective equipment (PPE), were experiencing very high workloads, and felt that their ability to effectively self-isolate was constrained (11). In the aged care sector, several academics and practitioners noted that adjusting the workforce model to lower casualisation in order to reduce the need for workers to work across so many homes, allowing for increased training, and ensuring that the workforce is supported to self-isolate if exposed to the virus were central to pandemic preparedeness (10, 12).

In all of these ways, the aged care and disability services sectors have faced significant challenges during the COVID-19 pandemic, many of which were pre-existing, but almost all of which were exacerbated by the pandemic conditions, and compounded to exacerbate health risks for clients. Understanding how organizations in these sectors adapted to the challenges posed by the pandemic, and the lessons that these adaptations offer for the future, is a critical element of ensuring that this crisis is not wasted and that the needs of older people and people with disability are better met going forward, both inside and outside of pandemic conditions. Accordingly, this paper presents the results of an exploratory study across three states (Western Australia, New South Wales and Victoria) examining, from service providers' perspectives, the nature of their adaptations and the innovations they adopted during the first year of the pandemic. The study examines which adaptations they want to carry forward, and the enabling factors that would be required if services wanted to continue what they suggest are improved practices.

### COVID-19 in Australia during the study period

Experiences of the pandemic were markedly different across the states included in this study throughout the first year of the pandemic (between March 2020 to March 2021). The variation in experience between states was due to varying frequency and severity of virus outbreaks and different state government policy approaches to virus containment (state governments are responsible for health policy in Australia). At 31 March 2021 (around the time data collection for this study concluded), the number of cumulative locally-acquired cases was 100 in WA (13), 2,179 in NSW (14), and 19,410 in VIC (15). Perhaps the most differentiating feature is the extent of the lockdowns experienced in each state, which were accompanied by significant service disruption. Between 30 June 2020 and 31 March 2021, Perth (WA) had been in lockdown for only 6 days and Sydney (NSW) 26 days (though only the Northern Beaches Local Government Area) (16), whereas Melbourne (VIC) had experienced three lockdowns for a total of 159 days (17). The lockdowns and other COVID-19 pandemic related restrictions had significant impacts on the aged care and disability services sectors. Accordingly, it is beneficial to contextualize the present study with information about the COVID-19 pandemic situation in Australia.

### Aged care and COVID-19 in Australia

Many of the COVID-19 pandemic government responses in Australia were targeted toward the aged care sector. At various stages of the pandemic, residential aged care facilities were not accepting visitors (aside for some exemptions on compassionate grounds), restricting the number of visitors per day, and/or limiting visit length. Personal protective equipment (PPE) was required for staff and visitors to residential aged care facilities and in-home care staff visiting older people in the community. Guidelines were also released for assessing the safety and necessity of home visits for home-based aged care, and adjustments were made to limit unnecessary in-person contact and visits. During lockdown conditions, the general public were only allowed to visit people outside of their households (including older people) if they were providing necessary care, and these visits were limited to two people maximum (i.e., the older person and the person providing care) (18).

Alongside concerns about physical health and care standards during the COVID-19 pandemic, substantial concerns were raised about social and emotional wellbeing among older people owing to the isolation and reduced interpersonal contact during the pandemic (8, 19). Further, older people were among the cohorts more likely to be digitally excluded as many businesses and services transitioned to online modes of delivery. At the start of the pandemic ~34% of Australians over 50 years of age (2.7 million people) either had low digital literacy levels or did not use digital devices or the internet (20). Some support measures were introduced by the federal government including funding for phone support lines for older people, extra staff to train volunteers to connect with people in aged care online or *via* phone, welfare checks, and mental health services (8). While states introduced various initiatives and funding to support the aged care sector, such as communities of practice (21), guidelines and advice (22, 23), most funding and direction for aged care services came from the federal government, reflecting their role in the aged care sector (8).

As the main funder and regulator of aged care, the Australian Government provided support to the sector that included online infection control training, provision of PPE, funding for “surge workforces,” onsite pathology for testing, staff retention bonuses, blanket payments for reimbursement of expenses incurred during the early stages of the pandemic, and funding of specific programs, particularly those providing food and meals. Additionally, visa rules were adjusted to allow increased working hours among international students employed in aged care and for working holidaymakers to work with single employers for more than 6 months if said employer was in a critical sector (including aged care) (24).

Consequently, aged care organizations had a number of interdependent priorities to balance, including physical vs. mental health of clients, the needs reported by clients vs. government funded support activities (and, in turn, organizational financial sustainability), and ensuring staff received the required training while also ensuring that services were adequately staffed.

### Disability services and COVID-19 in Australia

The disability services sector in Australia is guided by a National Disability Strategy and National Disability Agreement, and responsibilities are shared between the Australian, state and territory governments. The Australian Government provides employment, income support, and funding for administering and regulating the National Disability Insurance Scheme (NDIS), which provides a budget to the proportion of Australians with disability with the highest level of support needs, to access services and supports. State and territory governments share responsibility for some aspects related to the NDIS and input into determining national policy directions, and each have disability plans for their jurisdictions (25). For the better part of the last decade, services have been funded and provided on an individualized basis, whereby individuals are allocated a budget and spend that budget on eligible services in line with their needs (26).

Reflecting the shared responsibilities across federal and state/territory governments for disability services, priorities and responses for the disability services sector to respond to the pandemic were set at Council of Australian Government (subsequently renamed National Cabinet) meetings. Measures included the extension of NDIS participants' plans, flexibility in the use of funding (e.g., to purchase additional assistive technology), shifts to phone rather than face-to-face NDIS planning meetings, retention bonuses and additional funding for the disability services sector workforce, and collaboration with national supermarkets to allow NDIS participants to receive priority home delivery of groceries (25). States and territories introduced jurisdiction-specific initiatives, for example, the Western Australian government introduced a home delivery service for essential items and an employment website for disability services sector jobs. The Victorian government funded a suite of new programs and program extensions (25).

The individualization of disability funding and services in schemes like the NDIS seeks to increase choice and control among people accessing services (27). In the case of COVID-19 (and other infectious diseases), the NDIS had supportive functions but also resulted in unintended consequences and risks. Specifically, the casualisation and decentralization of the disability services workforce hindered information and education provision (e.g., about hygiene and infection control procedures) and meant that workers came into contact with many clients, and the “per service” funding model discouraged skipping of non-essential services or service components (e.g., to reduce COVID-19 exposure risk) (28, 29).

## Method

Given the exceptional circumstances of the COVID-19 pandemic and the multitude of different ways it impacted organizations in the aged care and disability services sectors, qualitative methods (semi-structured interviews with organizational representatives) were employed to allow us to explore the adaptations and experiences of organizations in detail. Ethics approval was granted by The University of Western Australia Human Research Ethics Committee (2019/RA/4/20/6461) and ratified by the committees at University of New South Wales and Swinburne University of Technology.

### Sample and recruitment

The study utilized a purposive sampling technique to ensure coverage of the sectors in terms of organization size; residential and community-based service delivery; and the types of innovations and adaptations expected (gleaned from media, social networks, involvement in other projects, and personal connections). The potential sample was determined as a team, and participating organizations were recruited through three strategies. First, we invited existing contacts that the research team had within relevant organizations. Second, we sought advice from peak bodies about which organizations had innovative pandemic adaptations and also requested that they advertise the project to their networks. Finally, we contacted organizations with no prior connection to the research team, but whose adaptations to the COVID-19 pandemic we had learned of. Organizations were contacted by e-mail to inform them about the project and invite their involvement or, in the case of peak bodies, interested organizational representatives were requested to contact us for further information.

Initially, we requested interviews with frontline staff, with the rationale that these staff would be most aware of adaptations that occurred “on the ground” and how they were received. However, most organizations nominated managers and/or executives to complete the interview. Organizational time constraints played a role in the sample; some organizations contacted were interested but too busy, some responded that they had not innovated during the COVID-19 pandemic, and others did not respond at all.

The sample comprised representatives from 26 aged care (*N* = 14) and disability services (*N* = 12) organizations across Western Australia (WA; *N* = 8), New South Wales (NSW; *N* = 11), and Victoria (VIC; *N* = 7). Three representatives were in exclusively frontline roles, 5 managerial with some frontline duties, with the remainder in managerial or executive roles. Three organizations provided primarily residential aged care, with the remaining organizations providing primarily in-home or community-based supports (e.g., day centers, appointment-based services, drop in services). Table 1 presents the distribution of organizations across the two sectors, by location and size based on the Australian Charities and Not-for-profits Commission classifications of annual turnover (*small* ≤ AUD250,000, *medium* = AUD250,000–1m, *large* ≥ AUD1m).

**Table 1:** Size, sector and location of participating organizations.

**Size**		**Sector**	
		**Aged care**	**Disability services**
	Small (annual turnover < AUD250,000)	**2** (1 NSW, 1 VIC)	**4** (1 WA, 2 NSW, 1 VIC)
	Medium (annual turnover AUD250,000–1 m)	**5** (4 NSW, 1 VIC)	**5** (2 NSW, 3 VIC)
	Large (annual turnover > AUD1m)	**5** (3 WA, 1 NSW, 1 VIC)	**3** (2 WA, 1 NSW)
	Private organizations	**2** (both WA)	–
	**Subtotal**	**14**	**12**

Organization size categories are determined by the Australian Charities and Not-for-profits Commission based the annual turnover reported in their 2021 Annual Information Statement. Two organizations could not be classified as they were private organizations and therefore not registered with the Australian Charities and Not-for-profits Commission.

### Data collection

Interviews were conducted between December 2020 and March 2021, primarily over Zoom and Microsoft Teams. Most interviews were one-on-one, with two organizations preferring to undertake a group interview with 2–3 organizational representatives and one interviewer. Interviews were recorded and the audio was transcribed verbatim.

A semi-structured interview schedule was developed based on two overarching questions: (1) what did services do differently during the COVID-19 pandemic? and (2) what do they want to do differently post-COVID-19 pandemic? The aim of the latter question was to explore the lessons from adaptations and innovations made in response to the COVID-19 pandemic (e.g., what worked well), as well as the impact of structural and contextual changes (e.g., funding agreements, client expectations) on the optimal functioning of organizations beyond the pandemic.

In addition to the above core questions, the interview schedule included a series of probing questions about why adaptations were made, facilitators and barriers to the adaptations, and enablers and obstacles for implementation of the adaptations on an ongoing basis. Background questions regarding organizational characteristics were also included, such as the nature of the services provided, the extent of impact of the COVID-19 pandemic on operations, and the role of the interviewee, to allow for consideration of how these factors interacted with organizations' responses to the COVID-19 pandemic, perceptions of the effectiveness of the responses, and attractiveness and feasibility of continuation of the responses.

### Data analysis

A researcher from each state (WA, NSW, and VIC) led coding of the transcripts from interviews undertaken in their respective states. All coding was conducted in QSR NVivo [RRID:SCR_014802], and coders adhered to a broad coding structure, with the following top and second level code categories:

- Organizational demographics.
  ° Interviewee role, main sector of operation, and services offered.

- Adaptations/innovations.
  ° Type/description, facilitators, and barriers.

- Post-COVID ambitions.
  ° Things to continue, things to stop, enablers, and obstacles.

**Figure 1:**
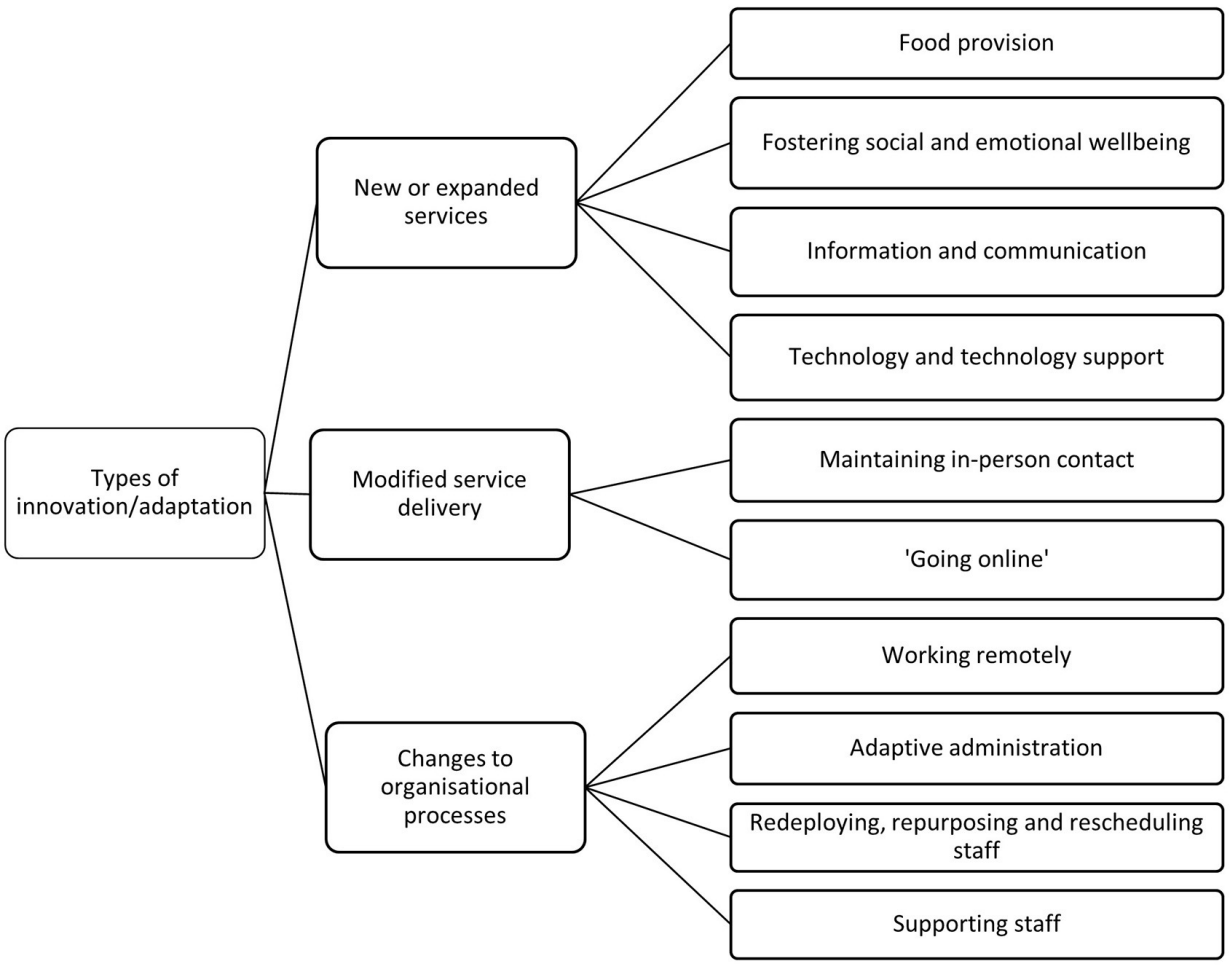
Coding structure of service adaptation and innovation themes identified.

**Figure 2:**
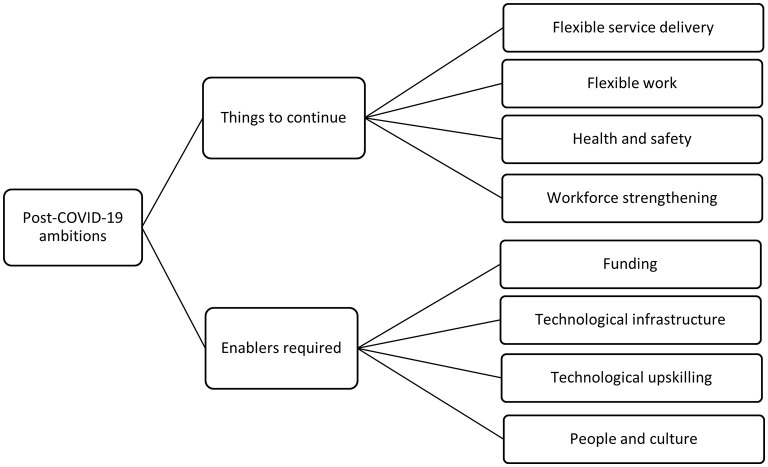
Coding structure of post-COVID-19 pandemic themes identified.

Each transcript was coded line-by-line, with each line assigned a descriptive code and placed under its relevant category in the broad coding structure. For example, a statement where an interviewee identified themselves as the CEO could be coded “Executive” underneath the second-level category of “Interviewee role.” The teams from each state discussed the results of the line-by-line coding to confirm agreement with the coding, and the full team discussed the themes that emerged in each state to identify overlap and points of difference, and thus the final set of themes. This paper focuses on the ways that organizations adapted to the COVID-19 pandemic, what they wanted to carry forward into the future, and what they needed to do so. The structure of these themes is presented in Figures 1, 2.

## Results

### RQ1: In what ways did services innovate and/or adapt during the COVID-19 pandemic?

Despite different COVID-19 pandemic experiences in each state, the aged care and disability services organizations in the three states had several commonalities in the ways in which they adapted to the pandemic. As illustrated in Figure 1 below, the adaptations comprised: (1) new or expanded services; (2) modified service delivery; and (3) changes to organizational processes.

#### New or expanded services

The first way in which organizations innovated in response to the COVID-19 pandemic was to introduce new services or expand their existing services to meet new or changing demand. As we elaborate, expansion of services involved: increasing the capacity (i.e., number of clients served); reducing or removing eligibility criteria for clients to receive the service; and/or expanding the scope of the service (i.e., increasing the types of services delivered within a program). In line with emergent client needs during the pandemic, the new and expanded services were mainly focused on: (1) food provision; (2) fostering social wellbeing; (3) information and communication; and (4) technology and technology support.

##### Food provision

Across sectors and states, the expansion or addition of food provision services was common among organizations in the study, in response to the perceived infection risks of grocery shopping, or difficulties getting to the shops during lockdowns, and grocery shortages. Several organizations created or expanded meal delivery services during the COVID-19 pandemic:

“*We would usually have criteria for people's eligibility to receive subsidized meals, but they wanted to support that for anyone. So it was Easter in 2020 and we put a call out... It was basically if you need food right now just give us a ring. So we doubled our meal delivery… over that period.”* ***WA8, disability***.

In addition to meal delivery, organizations introduced food boxes. A major element of food deliveries was the introduction of customized grocery services where organization staff consulted with people to ensure they received items they actually needed, rather than delivering generic boxes of food:

“*Because we had the* [Commonwealth government] *funding, we thought we'd make this a really good quality home delivery. We're not just providing pantry sort of shelf stable stuff. So we created a box that had a good variety of kitchen essentials, as well as adding in a kilo of fresh fruit and a kilo of fresh vegetables, some eggs and some bread… So as our workers were talking to people about what they needed* [when they rang up to book the delivery]*, they were able to add those things onto their order… The feedback that we've received from clients receiving those boxes has been—it's blown my mind… just the appreciation of being able to access the things that they needed… not just the generic boxes of food that they may or may not use, but really helpful items, in a timely manner.”* ***NSW1, aged care***.

Several aged care organizations also provided grocery shopping services to clients who were living in the community and were unable to, or hesitant about, going to the grocery store:

“*All* [clients] *had to do was leave the list outside. We would organize and tell them what time the support worker was coming, they would leave the list outside, and then it will all get done for them.”* ***VIC2, disability***.

“*We recognized that there was a digital divide for a lot of our clients where they didn't necessarily have access to either a smartphone or an email address to receive the* [supermarket] *e-vouchers that we were offering and so we came up with a system where we would provide a home delivery. Coles and Woolworths and IGA* [major supermarket chains] *were all offering some sort of home delivery service, but it was really, really difficult to access them…”* ***NSW1, aged care***.

In response to shortages in grocery stores and difficulties in getting to the shops among both clients and staff, one aged care organization started an ad-hoc “pantry” service:

“*If a client or a staff member was running out of food because they were not able to go to shop, then actually we bought a lot of food here. Was like a pantry really”* ***WA5, aged care***.

##### Fostering social wellbeing

Social isolation and stress were of particular concern among marginalized people during the COVID-19 pandemic (30–32). Organizations in the aged care and disability services sectors dedicated significant effort to fostering the social wellbeing of their clients. Particularly common were exercise classes and activities to facilitate socialization, fight boredom, and provide a sense of purpose. Several of these initiatives were undertaken digitally:

“*We had digital Zumba, we had digital cooking classes, where we had a worker who was preparing food, and we had clients at home watching it on their tablets, doing exactly the same for themselves.”* ***WA2, aged care***.

“*Our* [swimming] *coaches, they got together and they did Zoom sessions* [of land-based exercises]. *So they had four one-hour Zoom sessions every week at different times so that a broad range of our swimmers were able to get onto Zoom… all the coaches were involved.”* ***WA6, disability***.

On the other hand, to accommodate the needs and preferences of their clients and to combat screen fatigue, several organizations bucked the trend of “going online” during the COVID-19 pandemic. Offline initiatives included the delivery of “care packages” containing essential items and wellbeing activities, such as arts and crafts and letter writing kits. Part of the importance of this approach was that the delivery itself was used as a social event (with appropriate distancing and protective equipment):

“*Things like crosswords and word searches and knitting, like knitting packs or whatever their interest was… if they were interested in, I don't know, cars, they might get a car magazine… They were tailored to each of the consumers.”* ***VIC4***, ***disability***.

“*Because when we were coming, we would give them* [clients] *a call, ask them to come out, but what was important* [was that] *our clients had to brush their hair, to dress up into something nice and walk. Because our concern was that staying at home, they're losing a lot of functions. They don't walk, they don't exercise.”* ***NSW10, disability***.

There was also the introduction of regular “welfare checks” and social chats over the phone, and the dissemination of mindfulness and wellbeing exercises:

“*We rang them every day, so we had a calling system of calling every single client, every single day, to make sure that they were all okay.”* ***WA2, aged care***.

“*We constantly communicated with our clients. So even though we weren't able to undertake home visits as we normally would… we started undertaking welfare checks.”* ***VIC2***, ***disability***.

Many aged care organizations spoke about implementing various technologies to enable clients to speak with their families, with the setup and administration managed by the organization's staff. While this was not an entirely new activity, it became a much more common, formalized, and organized function of services during the COVID-19 pandemic:

“*So* [before COVID-19]*, if a resident needed support with* [communicating online with their family]*, we would help them and set it up for them or help them with their device or a family member might* [do so]. *But it was very ad hoc. We didn't have schedules around it. It was primarily also for residents with families overseas… But it just became much more formalized when this* [the pandemic] *took over and the numbers went up, because a lot of those families that wouldn't have used that in the past would just come and use it.”* ***NSW4, aged care***.

In the community, some in-home support workers usually responsible for personal care for aged care clients were also tasked with helping clients use technology to talk with family and friends.

##### Information and communication: Innovation through different channels

Unsurprisingly, given the high level of uncertainty brought about by the COVID-19 pandemic, communication of relevant information to clients, their families, and staff was a common area of innovation. Increased internal and external communication through various printed and online mechanisms occurred for almost all organizations, particularly to increase awareness and convey public health information and operational changes for services:

“[We had] *a lot of communication with the families. There was several* [communications used] *per week and during the height of it, there was almost daily contact with the families, telling them what they could do and what they couldn't do. How their relatives were being looked after, etc.”* ***WA4, aged*** ***care***.

“*The first part* [of the newsletter we made] *for the old folks* [was so that] *they know what is happening, talking about what's happening with Australia, within Australia, within WA, how we go, what it is the government's ruling and all that so they are aware of that. The second part, actually it is what* [the service] *is doing during this period of time.”* ***WA1, aged*** ***care***.

“*We had some contacts in hospitals, emergency departments. And we asked them to do videos for our members about how to don and doff PPE, so that we could share that. That sort of thing people were really quite in need of.”* ***NSW7, disability***.

In addition to standard printed and online materials, some organizations produced short videos that were emailed to clients and were posted on social media, other organizations started newsletters and/or bulletins that were mailed or emailed to clients. One organization set up a Google Meet room that was open during business hours for staff (and clients, by invitation), and another organization started a text-messaging bulletin.

Most organizations agreed on the need to increase communication efforts for continued effective operation, however the details of communication modes, delivery and purpose highlighted communication as innovation. For example, some organizations noted that information overload was common during the COVID-19 pandemic. Accordingly, they reflected that assessing the necessity of communication was required, and adjusting the frequency and size of communication in response, was part of how they innovated:

“*So, we started to disseminate a lot of that down into little bite size, you know, two or three lines at a time, so that it was more easily absorbed—we were trying to sort of balance everything, and bring a sense of normality to what we were doing.”* ***WA2, aged*** ***care***.

“*At the beginning with this updated newsletter, we send out twice a week at the beginning at the height of COVID… and then later we have it once a week, and later we have it fortnightly.”* ***WA1, aged care***.

Other organizations sought to address and reframe the public discourse circulating in the media about their clients and about social distancing:

“*A lot of the things that people said about elderly people during the pandemic, it's about they are very vulnerable and so forth and maybe physically they are, but they are also resilient. Especially our clients who are refugees of the Second World War and communism… They went through much worse than lockdown in the pandemic. So my CEO really highlighted that and said, “Look, we will look after you. We will keep you safe. But we also rely on your wisdom and resilience because we have not done this before.””* ***WA5, aged*** ***care***.

“*We really didn't like the phrase “social distancing” because we're very much a human connection organization… So we had an email banner… about physical distancing, human connection.”* ***WA8, disability***.

Accessibility of information was also a significant issue. For example, an important gap in the availability of COVID-19 information in different languages was noticed by organizations which service culturally and linguistically diverse communities. These organizations utilized the skills of their multilingual staff to rapidly translate and/or develop and disseminate important information to their clients:

“*Because there was a lot of confusion among many clients, because most of the information was in English and the clients were getting information from different places and there was sometimes even conflicting information. So… we decided to go up on newsletters in their own language.”* ***NSW3, aged*** ***care***.

“*We make sure that we have that done in three languages, it is English, Chinese, as well as Vietnamese. So to make sure that they know and they understand.”* ***WA1, aged care***.

In disability services, accessible information provision was about ensuring clients were able to access reputable health information in formats accessible to people with a range of disability-related support needs (e.g., Easy Read and Auslan [Australian Sign Language]), especially information that was specific to how people with disability should seek support regarding COVID-19 protections:

“*We started collating some of the resources that were more useful, informing each other of the responses from Health* [Department]. *Just started doing a load of outreach, putting it through our communications, popping them on our website and sharing them.”* ***NSW7, disability***.

“*We were also creating social stories*^1^ *and making that available to teams and we had posters that were there.”* ***NSW10, disability***.

##### Technology and technology support

Naturally, not all clients were adept at technology use at the outset of the pandemic. Accordingly, innovations related to online initiatives included support for clients to use technology. Some aged care and disability services organizations, for example, acquired tablets and devices:

“*A lot of clients that had never previously had tablets, or used the* [organisation's] *consumer portal, during COVID, migrated to that portal. So with the help of Apple, we offered them iPads, and we offered them training, so that they could basically manage and control their own services, for themselves.”* ***WA2, aged care***.

“*So what we had to do was get the devices all set up, same model, so we could pre-set them all up with Apple IDs and logins and things for someone, so they could just start using it, so brand new, ensuring privacy.”* ***NSW6, aged care***.

Common across organizations was the provision of wrap-around support and education for using new devices and virtual services such as Zoom, going beyond just setting it up. Support for using devices (and technology lessons in general) was as important, and in many cases more important, than providing the devices themselves. In some cases, existing support workers in aged care and disability services were tasked with providing this kind of technological support in addition to their usual roles:

“*We sent out our support staff to teach the seniors how to do video conferencing every single week for 2 weeks, they had like a hardcore Zoom training by our staff. And by the second, third week, they actually booked a Zoom meeting and they actually had one and it was such a big thing in the office.”* ***WA5, aged*** ***care***.

At least three organizations specialized in providing a suite of such lessons to clients, including seniors. Lessons ranged from simple teaching of device functionality to encouraging clients to be confident using technology and to be creative about exploring which programs and apps to use:

“*In Victoria and in New South Wales, we worked very, very quickly and we placed orders for iPads and we scaled up our staff around training…* [the] *bundle which would provide our clients with an iPad, with all set up, everything organized for them for someone to go* [in]*, and also for them to be trained. So we also put them* [clients] *through training as well.”* ***VIC2, disability***.

Notably, across the organizations, technology was used for a range of service functions, including fostering social wellbeing and social connections, as discussed earlier, but also for telehealth and service assessments.

#### Modified service delivery

Another way in which organizations innovated during the COVID-19 pandemic was by modifying or adapting their service delivery. These innovations can be broadly categorized into (1) maintaining face-to-face contact and (2) “going online.” It is important to contextualize modifications to service delivery in relation to the different restrictions in place in each state. For example, while WA and NSW organizations could operate some non-essential services in person by, for example, reducing group sizes, VIC organizations had to keep non-essential services online.

##### Maintaining face-to-face contact: Being creative

Many services offered by the aged care and disability service organizations require in-person contact. Therefore, a significant area of innovation was modifying the way in which services were delivered face-to-face in order to maintain service delivery and adhere to COVID-19 pandemic restrictions and guidelines. One disability service organization was able to continue face-to-face training and education but in smaller groups which they called “Pods” who rotated in visiting an onsite service hub. This change in the way face-to-face services were provided limited the risk of community transmission by shrinking contact to smaller groups:

“*Not everyone wanted to go online which was a challenge in itself, lots of people wanted to just stay in the hub. So we chose to do a partial closure where we did a rotation, so everyone had a turn, if they opted in, had a turn once a fortnight of coming into the hub for practical work experience. Then other than that they would be online with us on their normal working days.”* ***NSW9, disability***.

Other approaches included simple changes, such as having support workers conduct home visits that did not require close physical contact from outside the house, in front of a window or at the front door. Additionally, organizations developed methods of determining whether face-to-face contact was indeed needed, instead of providing it routinely. For example:

“*It* [the piece of paper] *had a smiley face on the green side, and it had a frown face on the red side, and that was for people to put in their windows of their homes… we had some volunteers, and some of our staff, drive by those houses every day, and have a look at the face in the window, and if the face in the window was green, we knew the client was okay. If the face in the window was red, the worker would stop, make a call to the office, the customer support team would ring the client and say, “Hey, you've got your red face up today, what's the matter? What can we help you with?” The client would say, “I”ve run out of milk, I have no bread, I need to go to the chemist, I'm not feeling well', or whatever it was, and then, the worker would be there, with PPE, to be able to assist them if needed.”* ***WA2, aged care***.

It is also important to note the flexibility that support workers took on in their roles. For example, if a client needed help with technology, or needed social support or assistance exercising, support workers would assist them. These activities were completed in addition to the worker's usual personal care and in-home assistance duties, reflecting the fact that support workers were often the only people allowed to be present in people's homes.

##### “Going online”

Going online was a typical way in which organizations modified their service delivery in response to the COVID-19 pandemic. In addition to the aforementioned online classes (e.g., exercise, cooking, and art) and social activities, many organizations took some elements of service delivery online. This included videoconferencing for consultations and other communication with clients; counseling/psychology appointments; service needs assessments; music/art therapy; visits with family (including foster care visitations); and for regular “office hours” in what had previously been “drop-in” services.

Videoconferencing was also used for providing social and educational programs to clients, for example, using online platforms for preparing clients for the workforce; and online social support and educational groups around domestic violence. Sometimes online content was also linked to physical resources that were sent out to clients:

“*Also we would send out a workbook; not for all webinars, but for at least 50%, where people just had time to work through a question in their workbook. So what they'd go away with at the end of the session is a bit of a plan of what they're going to do.”* ***VIC3, disability***.

#### Changes to organizational processes

Another area in which organizations innovated in response to the COVID-19 pandemic was through changes to organizational processes. Many of these changes were brought about by necessity, but several organizations were proactive, and others used the required adaptations as a springboard for further innovation. Changes to organizational processes fell into the themes of: (1) working remotely; (2) reducing bureaucracy; (3) re-deploying, repurposing and rescheduling staff; and (4) supporting staff.

##### Working remotely

As was common across industries, the COVID-19 pandemic saw a shift to working remotely for many functions of aged care and disability service organizations. The shift to remote working was a new practice for many of the organizations in the study for whom face-to-face was the primary mode of service delivery:

“*Community services—it's a very traditional way of doing things, and working from home was always frowned upon in community services, but I think the pandemic has taught us that working from home can make us work more effectively.”* ***NSW8, disability***.

“*COVID was actually really helpful for us because it decamped people that would never have been* [willing to make the shift]…* It would have been very hard—big change management, big resistance—to decamp. So we closed the* [suburb] *office… We were like let's seize this opportunity.”* ***WA8, disability***.

As in many other sectors, rapid adaptations to working at home gave rise to some physical and mental health and workplace safety concerns among staff. However, the organizations expressed how they came to appreciate the benefits of remote work for their workforce more generally, for example, reducing time spent traveling to meetings or better including staff members at satellite sites in regional and rural areas in meetings at metropolitan-based headquarters.

##### Adaptive administration

The quick onset and rapidly changing nature of the COVID-19 pandemic meant that organizations had to be agile in order to maintain services and supports for clients. Organizations reported they had developed new accountability structures for new service types and authorized new administrative processes that would not have occurred in the usual course of operation. Other organizations spoke about providing employees with decision-making frameworks or principles to empower them to make decisions (e.g., funding approvals) in a timely and efficient manner, rather than requiring them to engage in a hierarchical approval process.

##### Re-deploying, repurposing and rescheduling staff

Changes in service demand led to innovation around staffing and personnel. Some organizations mentioned either new staff joining their organization or the implementation of new structures and roles within their organization to assemble existing staff into roles tailored to pandemic needs. Examples included increasing the hours of staff with mental health qualifications; assembling inter-disciplinary teams to deal with complex problems arising for individual clients; and assembling a new management team dedicated to new COVID-19 management and resourcing:

“*We made sure we had a COVID-19 resource team that we assembled and there were seven of us that our job was… to work out what our strategy was going to be and how we deployed it and to just stay on top of everything, all communications that came out from NSW Health, so specifically for NSW, but also anything that came out from the Department of Health* [Commonwealth government]*.”* ***NSW10, disability***.

Other organizations made contractors employees of the organization so that they could continue to work and/or work more hours; introducing staggered rosters so that fewer people would be in the office at any given time and/or so teams did not interact (to minimize infection risk); and increasing the amount of hours that employees were working to minimize their need to be employed in another organization or role that may place them at higher risk of cross-infection between organizations or departments.

Provision of PPE and training around how to use it was a common theme in organizations that maintained face-to-face service delivery. Some organizations hired infection control specialists and others had staff from clinical teams train the other staff on the correct use of PPE. Several other organizations supported staff to undertake COVID-19 training that was required for their role, such as Commonwealth aged care and disability services training and training on child protection during the COVID-19 pandemic.

##### Supporting staff

In light of the increased stress placed on staff and the loss of in-person office interactions, debriefing sessions, and check-ins, several innovations during the COVID-19 pandemic related to supporting staff.

“*So where they were used to being together in an office and supporting each other, debriefing after they'd had a difficult client, throwing ideas “round, coming up with solutions, they didn't have that on-the-spot sort of connection anymore. So creating virtual meetings on a really regular basis allowed that connection to continue.”* ***NSW1, aged care***.

The prioritization of staff wellbeing is important to note as it reflects organizations' priorities during the COVID-19 pandemic, and because well-supported staff were essential for the success of the other innovations organizations undertook.

“*We said from day one and it was consistently messaged by our CEO all the way down…: “You come first. Work comes second. We will work it out together and you're fully supported.” And we're consistent in that message. Whatever they needed to be able to work from home, we supported them to do it. Sometimes that meant they went into the office and just totally stripped it bare from all cords, tables, chairs, whatever they needed to have their homes set up; us paying for their personal mobile bills if they couldn't get a work mobile quick enough. Whatever it is that they needed, we organized it.”* ***VIC6, aged care***.

Where staff did shift to remote working, most organizations spoke about putting processes in place to ensure they maintained very regular and intensive contact and support opportunities with staff, usually conducted online. Innovations included efforts to facilitate working from home, as well as teambuilding and social support activities, including virtual coffees, after-work drinks and trivia nights, and various formal and informal check-ins.

### RQ2: Which innovations/adaptations do organizations want to carry forward beyond the COVID-19 pandemic?

This section looks forward to how organizations would like to continue in service delivery post-COVID-19 pandemic and what would be required to support these changes in the long term.

It is important to note that at the time of writing, the COVID-19 pandemic remains a dynamic and evolving situation and it is not clear exactly when “post-pandemic” will be, nor what it will look like. Further, at the time of data collection (end of 2020 to the beginning of 2021), the outlook for the pandemic was optimistic. Nevertheless, a key aim of this research was to investigate which service innovations developed in response to the crisis phase of the pandemic brought about valuable learnings that could be integrated with or improve the usual suite of services. Accordingly, we asked participants to reflect on how they would like to operate in a post-COVID-19 pandemic environment. Though participants did not anticipate further widespread outbreaks leading to severe lockdowns, they responded with what they hoped for in terms of sustained changes and what they need to achieve this. As can be seen in Figure 2 below, a number of key themes were classified as (1) activities and practices to continue, and (2) requirements for continuation.

#### Activities and practices to continue

While the activities and practices that participants wanted to continue were nuanced and specific to their organizations, they could be categorized into themes, namely: (1) offering flexibility in service delivery and work arrangements; (2) maintaining health and safety procedures; and (3) staffing innovation.

##### Flexible service delivery and models of work

Many organizations reported that the COVID-19 pandemic shifted the attitudes of their clients and staff alike toward engaging in work and service delivery online. While a complete shift online was not desirable nor possible for any organization, there was definite appetite for online methods and opportunities for increasing safety, inclusivity, and accessibility of services that promoted social participation:

“*In terms of social group gatherings and outings, I think a lot of clients previously would not* [have attended]…* I think they found it to be a little bit of a hassle or they need extra support with getting in and out of a car and all of that sort of stuff, who actually shied away from wanting to do it as frequently as you can*, [compared to] *using virtual groups.”* ***VIC2***, ***disability***.

“*Whereas everything has always historically been delivered person-to-person, I think what we'll see is a more blended format of choice and control that starts to come out, you know? So, I may choose not to go to the center today, because I'm feeling a bit off, but I might choose to put a camera in the center, so that you can actually dial in and still participate, but you're just not physically there.”* ***WA2, aged care***.

“*The flexibility around service accessibility… where possible we would like to continue providing the virtual platforms because it saves traveling time. If* [clients] *are not able to attend the services for whatever reason they can still enjoy the services without having to travel.”* ***NSW5, aged care***.

Other service activities that organizations hoped to continue delivering flexibly included: family-client communication in residential aged care facilities, telehealth, and educational services.

With respect to models of work, flexible working options were also attractive to organization staff. For some, this included continuation of working from home for many staff at least part of the time. Others, in both the aged care sector (WA3) and the disability services sector (NSW10), intended to include video conferencing as an ongoing option for senior staff meetings and in clinical settings. Increased productivity, reduced commute times, and reduced costs were particular factors in favor of flexible work for NSW organizations:

“*I think most people enjoy that flexibility… it will also make us more productive, because if I don't have to go to* [suburb 1] *at nine o'clock, knowing that at 12, I have to be at a meeting in* [suburb 2]*, I can do my work from home, and then go to the meeting from here, you know? So, I think we need to continue to drive that way of doing things.”* ***NSW8, disability***.

Individual choice and control about how to engage with the organization was a central driver and consideration in offering flexibility in service delivery and in working conditions to clients and staff, respectively. Organizations recognized that a “one size fits all” model would not work for clients or staff, and consistently emphasized the need for flexibility in line with people's needs and preferences. Further, organizations acknowledged that these needs and preferences are subject to change. Thus, remote working and blended service delivery was not solely the innovation organizations intended to sustain beyond the COVID-19 pandemic. Instead, it was the ability to offer clients and staff options that worked best for them, at any given time and for any particular reason. For example, not having to cancel appointments or take a day off when feeling slightly under the weather; being able to work from home if a meeting is closer to home than the office; or being able to attend in-person when feeling in need of personal contact.

##### Health and safety procedures

Many organizations wanted to continue health and safety procedures, such as normalizing some infection control protocols, both for best practice reasons and for preparedness for any future COVID-19 outbreaks or related disruptions. This included continued use of PPE (WA4; aged care), maintenance of physical distancing protocols (WA6; disability, NSW1; aged care), continued infection control and hygiene procedures (NSW5; aged care), and changes to building design (such as smaller and “cosier” settings; WA4; aged care). Other examples included retaining infection control specialists and keeping teams formed during the COVID-19 pandemic (often comprising existing staff) *in situ*.

##### Staffing

Staffing innovation post-COVID-19 pandemic focused on retention and recruitment. Retention and recruitment goals were about maintaining and expanding the skill mix that they had achieved through recruitment of new staff or restructuring of teams during the heightened pandemic conditions. In addition to retaining existing staff, several organizations saw the need to continue growing their workforce of paid employees and volunteers, and were looking into funding sources, online traineeships, and other opportunities to drive recruitment.

Finally, another aspect that organizations wanted to continue was the rostering of staff to minimize their infection risk. For example, an aged care organization (WA4) indicated that they would be restructuring their staff rostering to offer more hours to staff who wanted them, in order to reduce the number of organizations that staff had to work at in order to derive sufficient income.

#### RQ3: What do organizations need to continue and/or expand innovations/adaptations introduced during COVID-19?

Organizations noted several requirements in order to continue beneficial activities and practices beyond the COVID-19 pandemic: (1) funding, (2) technological infrastructure, (3) technological upskilling, and (4) people and culture.

##### Funding

Funding and funder flexibility were significant requirements for organizations in moving forward after the COVID-19 pandemic. During the pandemic, organizations, their employees, and some clients were being supported by government funding to help them through the crisis. The funding enabled the sector to quickly adapt and innovate, but it was never intended to sustain organizations nor their innovations over a long period of time.

“*If those* [client] *numbers increased to the level that we were funded to deal with, we would need to look to see if we could have and find alternate funding sources for this… obviously it's very difficult to get a new service funded.”* ***WA1, aged care***.

Questions about the compatibility of traditional funding administration models with organizations' new requirements, realized during the COVID-19 pandemic, was also a key issue in the aged care and disability services sectors. One organization (WA4) noted that funding of aged care facilities was still based on the older “hotel-style” building, and that governments needed to adapt that funding model to be more in line with dementia best practice and COVID-19 safety. Another aged care organization who also provided disability services (WA2) talked about the tensions between the free market approach of the individualized funding model used by the NDIS, and the excessive constraints they felt service providers were under, rendering them uncompetitive in the newly created market.

Some organizations who delivered disability services also felt that the diversity of need and preferences among clients, and the success of different funding models during the COVID-19 pandemic, should give rise to more flexible funding:

“*Whereas I think one of the things government really should be doing is recognizing the diversity of needs and preferences and abilities and interests and actually ensuring that there are different models in the sector solving problems in different ways and then, in the context of a particular market challenge like the pandemic, it allows more flexibility to respond.”* ***VIC5, aged*** ***care***.

“*It probably will also be helpful if NDIS makes some changes around how the budgets are built… they're proposing that it should go to the two categories; fixed and flexible, that would be amazing. If we could just have fixed and flexible, that would be great.”* ***WA7, disability***.

##### Technological infrastructure

Technological infrastructure was identified by several organizations as a requirement for them to continue and/or expand the innovations they undertook during the COVID-19 pandemic. For example, a disability service organization (WA7) mentioned the need to streamline technological platforms and align them to their new relationship-oriented model of service, and another disability service organization (WA8) talked about the need to move everyone to laptops to facilitate hot desking and remote working.

The need for technological infrastructure among organizations also included their ability to provide devices to their clients. One organization, after seeing the utility of iPads, was going to seek to introduce Apple watches into service delivery:

“*When we really started looking at it* [Apple watches]*, for the individuals that have—they have now moved into using an iPad for telehealth appointments. We have clinical nurses and they're doing video calls, etcetera. They're having telehealth appointments with their GP or with a physiotherapist. All of that is happening using their iPad… They can have more and more control of themselves and their health and their wellbeing—all of that sort of stuff.”* ***VIC2, disability***.

In order to secure the desired technological infrastructure, organizations discussed continuing relationships with technology providers and finding sources of funding to purchase devices.

##### Technological upskilling

In addition to acquiring and/or upgrading technological infrastructure, many organizations identified the need to upskill and support staff and clients in technology use. As the COVID-19 pandemic highlighted technological barriers faced by certain cohorts, the sustained use of innovative methods such as online access to services is dependent on the removal of those barriers. Organizations who work with these demographic groups require appropriate support based on their needs. This was highlighted by one organization who worked with culturally and linguistically diverse clients who also have a disability and access the NDIS.

“*I think we have a responsibility to kind of spend a bit of time, and investment and resources… on training NDIS participants on how to navigate the* [NDIS] *portal… on how to use online technologies to be supported… to remind them that these things exist, and not be afraid to try it out… but we can't do it for free, basically, because that's resource-intensive. We need to be able to apply for grants, so that we can do it in addition to the support coordination, and all the other assistance that we provide.”* ***NSW8, disability***.

As noted earlier, the pandemic saw staff take on tasks such as technological troubleshooting and training clients to use technology, which were well out of their usual duties and, often, outside of their knowledge and skills. As one organization noted:

“*… you've got staff that have got no idea and they're there because they're skilled* [at personal care]. *Their knowledge is providing disability supports but not IT links and connections.”* ***VIC4, disability***.

##### People and culture

For long-term sustainable change or innovation to continue, human resources are critical. The COVID-19 pandemic has demonstrated the significance and practical utility of technology in various forms. However, to operate or use technology, organizations still need sufficient staff and volunteers. During the pandemic, while organizations had been able to adapt or pool staff skills together or get external volunteer support to provide innovative service to their clients, it was only a short-term measure. Sustaining such mechanisms over a long period can only be done with more human resources and support from volunteers, or by recruiting people with technological skills who can help the organization and their clients' needs.

Staff willingness to participate in ongoing cultural change and the need to foster it were also key requirements for post-COVID-19 pandemic initiatives. An aged care organization (WA1) talked about needing to “bring everyone in” and flattening the hierarchy to “appreciate each other's strengths no matter what position you are in.” They also discussed the need to develop a culture of adaptability to change:

“*I would say we are learning, all the time we have to adapt to new changes… Normally everyone, including clients, everyone would like to remain in their comfort zone, but now we have to motivate everyone* [toward] *understanding why there's a need to get out from their comfort zone. We may have to change, it's the new normal.”* ***WA1, aged care***.

One disability service organization (WA7) talked about personality differences between staff and the need to provide training that is aligned with their individual needs. Another disability service organization (WA8) identified a need to combat staff isolation with a more remote working model by developing strong teams within local “hubs.” Further, recognizing the blurring of the lines between work and home that occurred during the COVID-19 pandemic, many organizations talked about the need to continue supporting staff both in the workplace and outside of it.

## Discussion

This paper presented a qualitative summary of the service adaptations and innovations of 26 aged care and disability services organizations across three states (WA, NSW, and VIC) in Australia during the early phases of the pandemic (roughly the first year). Three key areas of innovation were identified: (1) new or expanded services; (2) modified service delivery; and (3) changes to organizational processes. Additionally, we explored the organizations' ambitions for maintaining beneficial changes during the post-COVID-19 pandemic era and the resources required to sustain these changes.

### Innovations and post-COVID-19 pandemic ambitions

Reviewing the findings of this study, it is notable that some innovations and adaptations were implemented to address anticipated risks identified at the start of the pandemic, such as initiatives to address loneliness and social isolation and the dissemination of accessible and understandable information about the pandemic (5, 7). On the other hand, some changes were in response to unforeseen pandemic impacts, such as the addition and expansion of food provision services to alleviate the impacts of panic buying and subsequent grocery shortages. Other adaptations fell somewhere in between, such as the provision of technology and technology support. While the need for additional technology was identified early, additional support was required for its implementation. The adaptive responses to both anticipated and more unanticipated challenges required organizations to enact both initial change at the outset and ongoing change to respond to the evolving situation. A striking feature of these adaptations was the level of direction provided by the clients. Food delivery services were provided with significant input from the client, such that they could request specific items in food parcels, make shopping lists, or select items from a community pantry. Social wellbeing activities were delivered online or offline based on the preferences of the client. Further, organizations developed unique ways for clients to let them know what they need, ranging from daily phone calls to displaying coded cards in their windows. Taken together, these adaptations highlight the importance of providing individualized support and empowering clients to make choices about their care.

Likely reflecting the sample, such that interviewees were mostly management and executives representing their organizations, several adaptations were related to organizational processes, such as the introduction of working from home, initiatives to provide instrumental and emotional support to staff, and reducing bureaucracy. Additionally, the Royal Commission into Aged Care Quality and Safety, alongside several academics and practitioners, raised concerns about the casualisation of the workforce and the risks this created in terms of virus transmission and staff shortages, with obvious and serious consequences for the health and wellbeing of clients (10, 12, 18). It is perhaps heartening to note that some organizations adapted their workforce policies and strategies to facilitate more job security and stability for workers, thus enabling workers to hold fewer jobs, reducing their risk of catching and broadly transmitting the virus, and lowering the organizations' risk of staff shortages and substandard care. Even more heartening is that some organizations reported that they wanted to carry forward these workforce strengthening measures into the future as part of their usual operations.

Another innovation that many organizations wanted to maintain was the provision of hybrid online/in-person service delivery. Most organizations approached the introduction of these hybrid arrangements as an opportunity to increase the level of choice and control clients had over their engagement in services. Face-to-face modes remain the preferred or standard mode of delivery, but online options could be available so that clients did not miss out on services if they were feeling unwell or had difficulties with transport, particularly for more discretionary services like social activities. Ongoing implementation of hybrid systems would help increase inclusivity, and mitigate the loneliness and social isolation often experienced by older persons and people living with disability (generally, and in pandemic conditions), without many of the risks of digital exclusion posed by going fully online (3).

The intent of many organizations to continue COVID-19 health and safety measures is interesting to note. On the one hand, it is unsurprising that health-oriented services and/or services that deliver supports to people who are at higher risk from infectious diseases in general are on-board with infection control measures. On the other hand, the more recent phase of the pandemic in Australia (and, indeed, much of the world) has been characterized a substantial push to return to “normal” pre-pandemic conditions (33). It would be interesting to see if organizations' resolve to continue to protect their clients from infection risks has wavered in response various political and cultural norms and expectations, or has expanded in light of evidence about the role of ventilation and good quality masks in reducing transmission of COVID-19 and other airborne viruses (34–36).

### Requirements for continuation

Several requirements for the continuation of adaptations made during the pandemic and, indeed, service innovations, were noted by organizations. Funding was a major requirement, particularly for organizations who needed to make large scale investments in technological infrastructure, and for organizations whose service offerings and client-base had expanded during the pandemic. Flexibility of how existing funding could be used, as well as more consistent and long-term funding, was another key enabler for maintaining ongoing innovation. Accountability is a key issue for Australia's (and many other countries') not-for-profit organizations, such that there is a need to demonstrate the difference they make with the resources they receive, and no consensus about the best way to do so (37, 38). This poses an interesting challenge in the context of both innovation and crisis—the flexibility around expenditure of funding necessitated by the pandemic allowed organizations to innovate, which (they believe) allowed them to better meet client needs. For this level of flexibility (and, thus, ongoing innovation) to be supported by commissioning practices on an ongoing basis, organizations and their funders will have to collaboratively determine appropriate accountability mechanisms that ensure that clients' needs are best served and organizations are supported to take the risks inherent to innovation (39).

Some organizations called for simplification of funding models, particularly around the budgets allocated to individuals, which were seen to be restrictive and, at times, hindering clients from getting what they want and need. This mirrors evidence from the client perspective, such that there are substantial differences in the utilization of individualized support budgets by socioeconomic status and type of need, attributed largely to differences in information (40, 41). Therefore, while individualized funding results in higher client satisfaction, quality of life and safety (42), there is inconsistency in these outcomes across cohorts because of systemic barriers to effectively accessing information and utilizing their individual budgets accordingly. Therefore, for organizations to be effective and clients to receive the services they need, the service system must be navigable (i.e., clients must have the information and flexibility to use their funding to acquire the services they need) and organizations must have adequate support to assist with navigation and the provision of services.

Additionally, several organizations reported they needed infrastructure and training to use the technology required for hybrid systems, as well as ongoing support for (and from) staff and volunteers to successfully maintain the changes. Accordingly, if technology is to be successfully used in service delivery, existing staff will need to be upskilled and/or new staff will need to be recruited into roles that specify technological support as duties. This, once again, will require additional funding or reallocation of funding if possible (e.g., if demand for some services is permanently reduced in a post-pandemic environment).

Finally, organizations noted that staff and organizational openness and adaptability to cultural change was critical to the continuation of innovation beyond the pandemic. Given longstanding issues around burnout among workers in the care economy (43, 44), which have only been exacerbated by the ongoing COVID-19 pandemic (45), efforts will need to be made to retain workers and look after their wellbeing. This has been recognized in Australia, with the federal government making a value case to the Fair Work Commission (key national industrial relations body) for a rise in care worker pay, and the government's recent Jobs Summit proffering many suggestions to retain and reduce pressure from workers, from waiving higher education debt to increasing the workforce (46).

### Limitations and future directions

This study is subject to several limitations. First, the 26 organizations were recruited using a purposive sampling method. While the sample was quite large for qualitative research and appropriate for our exploratory approach, the experiences reported here should not be interpreted as representative of all organizations in the Australian aged care and disability services sectors. Participating organizations were specifically selected because of their successful innovation and adaptation of services to capture learnings to inform the sector, and the organizations who agreed to take part may have been more likely to be confident about their experience of innovating, thus the findings should be interpreted in that context. Noting that the scope of the current study focused only on organizations who were able to implement the innovations identified, future research should investigate the capacity for adopting these innovations (in terms of resources and organizational structure) across a wider range of organizations within the aged care and disability services sectors.

Further, the findings are from organizations in Australia, which had, at the time of the study, had a mild experience of the pandemic in terms of cases and deaths. Therefore, the nature and extensiveness of adaptations reported may differ from organizations in countries that were managing higher risks of infection in addition to COVID-19 related restrictions, and almost certainly differ from organizations in lower-income countries that did not receive a similar level of government financial support as Australian organizations. Importantly, however, there were many similarities across organizations in different states despite pandemic experiences ranging from having one of the lowest rates of community transmission in the world (i.e., WA) to having one of the highest levels of lockdowns and associated restrictions in the world (i.e., VIC). This is likely largely due to context, such that many restrictions were put in place nationally and participating organizations delivered mostly essential services and were thus able to continue many services regardless of restrictions, albeit in an adapted way. However, the convergence of themes does suggest that the innovations we have reported here have applicability beyond pandemic conditions and could be adopted in a range of regions, sectors, and contexts.

The COVID-19 pandemic context has changed rapidly in Australia since the study period, such that cases and community transmission are widespread and deaths have regularly exceeded 50 nationally per day, under a so-called “living with COVID-19” strategy. Despite the general ability of COVID-19 vaccines to prevent severe illness and hospitalization, older people, people with disability, and those that are immunocompromised are still at high risk of adverse outcomes, which has been reflected in hospitalization and death statistics. Accordingly, organizations' priorities may have evolved and, indeed, new adaptations and innovations are likely to have emerged since the data collection for this paper was completed. However, as we have noted, many adaptations reported by organizations in the earlier stages of the pandemic will remain relevant and have utility beyond pandemic applications because of the strong focus on maintaining wellbeing, increasing social inclusion, highlighting increased choice and control for clients, and reducing infection risk in both clients and staff.

Finally, the innovations and associated efficacy reported in this paper have been derived only from the perspective of the organizations and have not been evaluated. Future research should include comprehensive assessment and evaluation of the effectiveness of innovations and adaptations that affect clients, and decisions to continue or discontinue them, that involves the views of service users. With particular regard to older persons and persons with disability, the heterogeneity of needs among these cohorts must also be considered.

### Conclusion

As we enter a new phase in Australia's pandemic response—and as our cultural understandings of risk shift once again—it is useful to reflect on the ways that these 26 aged care and disability service organizations adapted so responsively and willingly to adverse early pandemic conditions. What was notable was the appetite for organizations to take on an unusually heavy burden of risk through embracing widespread change (and consequently the rapid staffing, administrative and operational shifts), while doing everything in their power to reduce physical health and psychological harms for the clients they served and the staff they employed. Indeed, the centrality of client and staff needs and the widespread accommodation of their preferences was perhaps the most striking feature of the organizations' responses to the pandemic. Accordingly, the innovations and adaptations that organizations indicated they wanted to carry beyond the pandemic were those that increased client choice and safety, such as maintaining online options for service delivery. However, it was noted that additional funding and flexibility of funding would be needed to support the infrastructure and training investments required to support ongoing implementation of adaptations. When aggregated, these experiences provide evidence of how organizations can adapt to widespread risk and change, and offer ideas of how other organizations in what have traditionally been face-to-face service roles can innovate in a widespread health crisis. Further, they point to opportunities for improvements to the rules surrounding the expenditure of funding allocated to organizations and individuals, accompanied by refined, collaboratively developed accountability mechanisms.

## Data availability statement

The datasets presented in this article are not readily available because the transcripts analyzed for this study are not publicly available due to the identifying nature of the content. Requests to access the datasets should be directed to ami.seivwright@utas.edu.au.

## Ethics statement

The protocol for this study was reviewed and approved by the University of Western Australia Human Research Ethics Committee and reciprocally approved by University of New South Wales and Swinburne University of Technology. The participants provided their written and/or verbal informed consent to participate in this study.

## Author contributions

AS, MA, MV, LK, PC, and AM undertook data collection. AS, PC, MV, and AM undertook the analysis. AS completed the first draft of the manuscript, each author undertook a round of writing and editing, and reconciled the edits for submission. All authors contributed equally to the design of the overall study and the data collection instrument.

## Funding

This research was funded by the Center for Social Impact Building Back Better research program.

## Conflict of interest

The authors declare that the research was conducted in the absence of any commercial or financial relationships that could be construed as a potential conflict of interest.

## Publisher's note

All claims expressed in this article are solely those of the authors and do not necessarily represent those of their affiliated organizations, or those of the publisher, the editors and the reviewers. Any product that may be evaluated in this article, or claim that may be made by its manufacturer, is not guaranteed or endorsed by the publisher.

## Footnotes

^1^Accessible format for people with intellectual disability or autism.

## References

[1] Rollston R Galea S. The Coronavirus Does Discriminate: How Social Conditions are Shaping the COVID-19 Pandemic. Harvard: Harvard Medical School Centre for Primary Care (2020). Available online at: http://info.primarycare.hms.harvard.edu/blog/social-conditions-shape-covid (accessed August 20, 2022).

[2] Finch WH Hernández Finch ME. Poverty and Covid-19: rates of incidence and deaths in the United States during the first 10 weeks of the pandemic. Front Sociol. (2020) 5:47. 10.3389/fsoc.2020.00047 33869454 PMC8022686

[3] Crawford A Serhal E. Digital health equity and COVID-19: the innovation curve cannot reinforce the social gradient of health. J Med Internet Res. (2020) 22:e19361. 10.2196/19361 32452816 PMC7268667

[4] Seifert A Batsis JA Smith AC. Telemedicine in long-term care facilities during and beyond COVID-19: challenges caused by the digital divide. Front Public Health. (2020) 8:601595. 10.3389/fpubh.2020.601595 33194999 PMC7649197

[5] Armitage R Nellums LB. The COVID-19 response must be disability inclusive. Lancet Public Health. (2020) 5:e257. 10.1016/S2468-2667(20)30076-1 32224295 PMC7270835

[6] Doyle L O'Brien J A. cacophony of protocol: disability services in the context of the Covid-19 pandemic. Irish J Sociol. (2020) 28:370–4. 10.1177/0791603520942681

[7] Aitken GE Holmes AL Ibrahim JE. COVID-19 and residential aged care: priorities for optimising preparation and management of outbreaks. Med J Aust. (2021) 214:6–8. 10.5694/mja2.50892 33296510

[8] Siette J Wuthrich V Low LF. Social preparedness in response to spatial distancing measures for aged care during COVID-19. J Am Med Direct Assoc. (2020) 21:985–6. 10.1016/j.jamda.2020.04.015 32674832 PMC7167563

[9] Government of Australia Royal Commission into Aged Care Quality Safety. Care, Dignity and Respect. Final Report (2021). Available online at: https://agedcare.royalcommission.gov.au/publications/final-report (accessed August 10, 2022).

[10] Carey G. The national disability insurance scheme and COVID-19: a collision course. Med J Aust. (2020) 213:141–e1. 10.5694/mja2.50690 32609380 PMC7361423

[11] Cortis N van Toorn G. The Disability Workforce and COVID-19: Initial Experiences of the Outbreak. Sydney: Social Policy Research Centre, UNSW Sydney (2020).

[12] Usher K Durkin J Gyamfi N Warsini S Jackson D. Preparedness for viral respiratory infection pandemic in residential aged care facilities: a review of the literature to inform post-COVID-19 response. J Clin Nurs. (2021) 2021:1–14. 10.1111/jocn.15863 34021650 PMC8242770

[13] COVID Live. Daily and Cumulative Cases by Source of Infection, Western Australia. Available online at: https://covidlive.com.au/report/daily-source-overseas/wa (accessed August 21, 2022).

[14] COVID Live. Daily and Cumulative Cases by Source of Infection, New South Wales. Available online at: https://covidlive.com.au/report/daily-source-overseas/nsw (accessed August 21, 2022).

[15] COVID Live. Daily and Cumulative Cases by Source of Infection, Victoria. Available online at: https://covidlive.com.au/report/daily-source-overseas/vic (accessed August 21, 2022).

[16] Australian Government Department of Education Department of Employment Workplace Relations. ECEC COVID-19 Timeline (2021). Available online at: https://www.dese.gov.au/covid-19/resources/ecec-covid19-timeline (accessed August 21, 2022).

[17] Lockdown Stats Melbourne (2021). Available online at: https://lockdownstats.melbourne/ (accessed August 21, 2022).

[18] Australian Government Aged Care Quality Safety Commission. COVID-19 Information. (2022). Available online at: https://www.agedcarequality.gov.au/covid-19-information (accessed August 21, 2022).

[19] Atkins M Baldassar L. COVID-19, Social Isolation Ageing: CSI Response. (2020). Available online at: https://www.csi.edu.au/media/uploads/csi_fact_sheet_social_covid-19_social_isolation_and_ageing.pdf (accessed August 21, 2022).

[20] Office of the eSafety Commissioner. Understanding the Digital Behaviours of Older Australians. (2018). Available online at: https://www.esafety.gov.au/sites/default/files/2019-08/Understanding-digital-behaviours-olderAustralians-summary-report-2018.pdf (accessed August 21, 2022).

[21] New South Wales Health. Aged/Care Aged Health. COVID-19 (Coronavirus) Communities of Practice. St Leonards: New South Wales Health (2022). Available online at: https://www.health.nsw.gov.au/Infectious/covid-19/communities-of-practice/Pages/aged-care.aspx (accessed August 21, 2022).

[22] Western Australian Department of Health. COVID-19 Information for Aged Care and Community Care Providers. East Perth: Western Australian Department of Health (2022). Available online at: https://ww2.health.wa.gov.au/en/Articles/A_E/Coronavirus/COVID19-information-for-Aged-Care-and-Community-Care-Providers (accessed August 21, 2022).

[23] Victorian Department of Health. Residential Aged Care Sector: COVID-19. Victoria: Victorian Department of Health (2022). Available online at: https://www.health.vic.gov.au/covid-19/aged-care-sector-covid-19 (accessed August 21, 2022).

[24] Grove A. COVID-19 Aged Care: A Quick Guide. Parliament of Australia Research Paper Series 2019–2020 (2020). Available online at: https://www.aph.gov.au/About_Parliament/Parliamentary_departments/Parliamentary_Library/pubs/rp/rp1920/Quick_Guides/COVID-19AgedCare (accessed August 21, 2022).

[25] Biggs A Storen R Corrigan N. COVID-19: Chronology of Australian Government Announcements on Disability Services. Parliament of Australia Research Paper Series 2019–2020 (2020). Available online at: https://www.aph.gov.au/About_Parliament/Parliamentary_departments/Parliamentary_Library/pubs/rp/rp2021/Chronologies/COVID19-DisabilityServices (accessed August 21, 2022).

[26] Carey G Malbon E Olney S Reeders D. The personalisation agenda: the case of the Australian national disability insurance scheme. Int Rev Sociol. (2018) 28:20–34. 10.1080/03906701.2018.1425084

[27] Meltzer A Dickinson H Malbon E Carey G. Why is lived experience important for market stewardship? A proposed framework for why and how lived experience should be included in stewarding disability markets. Evid Policy. (2021) 17:335–47. 10.1332/174426421X1614271494699623599727

[28] Yates S Dickinson H. Navigating complexity in a global pandemic: the effects of COVID-19 on children and young people with disability and their families in Australia. Public Admin Rev. (2021) 81:1192–6. 10.1111/puar.13352 33821041 PMC8014242

[29] Dickinson H Carey G Kavanagh AM. Personalisation and pandemic: an unforeseen collision course? Disabil Soc. (2020) 35:1012–7. 10.1080/09687599.2020.1772201

[30] Smith ML Steinman LE Casey EA. Combatting social isolation among older adults in a time of physical distancing: the COVID-19 social connectivity paradox. Front Public Health. (2020) 2020:403. 10.3389/fpubh.2020.00403 32850605 PMC7396644

[31] Lund EM Forber-Pratt AJ Wilson C Mona LR. The COVID-19 pandemic, stress, and trauma in the disability community: a call to action. Rehabil Psychol. (2020) 65:313. 10.1037/rep0000368 33119381

[32] Patel SS Clark-Ginsberg A. Incorporating issues of elderly loneliness into the Coronavirus Disease−2019 public health response. Disaster Med Public Health Prepar. (2020) 14:e13–4. 10.1017/dmp.2020.145 32379016 PMC7251282

[33] Baxter N MacIntyre CR. Cutting COVID isolation and mask mandates will mean more damage to business and health in the long run. In: The Conversation. (2022). Available online at: https://theconversation.com/cutting-covid-isolation-and-mask-mandates-will-mean-more-damage-to-business-and-health-in-the-long-run-189862 (accessed September 4, 2022).

[34] Morawska L Ayoko GA Bae GN Buonanno G Chao CY Clifford S. Airborne particles in indoor environment of homes, schools, offices and aged care facilities: the main routes of exposure. Environ Int. (2017) 108:75–83. 10.1016/j.envint.2017.07.025 28802170

[35] Deng W Sun Y Yao X Subramanian K Ling C Wang H. Masks for COVID-19. Adv Sci. (2022) 9:2102189. 10.1002/advs.202102189 34825783 PMC8787406

[36] Martinelli L Kopilaš V Vidmar M Heavin C Machado H Todorović Z. Face masks during the COVID-19 pandemic: a simple protection tool with many meanings. Front Public Health. (2021) 8:606635. 10.3389/fpubh.2020.606635 33520918 PMC7838459

[37] Gilchrist DJ Simnett R. Research horizons for public and private not-for-profit sector reporting: moving the bar in the right direction. Account Finan. (2019) 59:59–85. 10.1111/acfi.12439

[38] Adams S Simnett R. Integrated reporting: an opportunity for Australia's not-for-profit sector. Aust Account Rev. (2011) 21:292–301. 10.1111/j.1835-2561.2011.00143.x

[39] Edelson DC. Balancing innovation and risk. Educ Des Res. (2006) 22:100–6.

[40] Malbon E Weier M Carey G Writer T. How personalisation programs can exacerbate socio-economic inequities: findings from budget utilisation in the Australian national disability insurance scheme. BMC Public Health. (2022) 22:1–2. 10.1186/s12889-022-13301-x 35501795 PMC9061231

[41] Devine A Dickinson H Rangi M Huska M Disney G Yang Y. “Nearly gave up on it to be honest”: utilisation of individualised budgets by people with psychosocial disability within Australia's national disability insurance scheme. Soc Policy Admin. (2022) 1–18. 10.1111/spol.12838

[42] Fleming P McGilloway S Hernon M Furlong M O'Doherty S Keogh F. Individualised funding interventions to improve health and social care outcomes for people with a disability: a mixed-methods systematic review. Campbell Syst Rev. (2019) 3:e1008. 10.4073/csr.2019.3 PMC8356501 37131462

[43] Dall'Ora C Ball J Reinius M Griffiths P. Burnout in nursing: A theoretical review. Human Resour Health. (2020) 18:1–7. 10.1186/s12960-020-00469-9 32503559 PMC7273381

[44] Rehder K Adair KC Sexton JB. The science of health care worker burnout: Assessing and improving health care worker well-being. Arch Pathol Lab Med. (2021) 145:1095–109. 10.5858/arpa.2020-0557-RA 34459858

[45] Çelmeçe N Menekay M. The effect of stress, anxiety and burnout levels of healthcare professionals caring for COVID-19 patients on their quality of life. Front Psychol. (2020) 11:597624. 10.3389/fpsyg.2020.597624 33329264 PMC7719786

[46] Australian Government. Jobs + Skills Summit Outcomes. Available online at: https://treasury.gov.au/sites/default/files/inline-files/Jobs-and-Skills-Summit-Outcomes-Document.pdf

